# MIL-88A Metal-Organic Framework as a Stable Sulfur-Host Cathode for Long-Cycle Li-S Batteries

**DOI:** 10.3390/nano10030424

**Published:** 2020-02-28

**Authors:** Almudena Benítez, Juan Amaro-Gahete, Dolores Esquivel, Francisco José Romero-Salguero, Julián Morales, Álvaro Caballero

**Affiliations:** 1 Departamento de Química Inorgánica e Ingeniería Química, Instituto Universitario de Investigación en Química Fina y Nanoquímica, Facultad de Ciencias, Universidad de Córdoba, 14071 Córdoba, Spain; q62betoa@uco.es (A.B.); iq1mopaj@uco.es (J.M.); 2 Departamento de Química Orgánica, Instituto Universitario de Investigación en Química Fina y Nanoquímica, Facultad de Ciencias, Universidad de Córdoba, 14071 Córdoba, Spain; q22amgaj@uco.es (J.A.-G.); q12esmem@uco.es (D.E.)

**Keywords:** Li-S battery, metal-organic framework, sulfur composite, polysulfides confinement

## Abstract

Lithium-sulfur (Li-S) batteries have received enormous interest as a promising energy storage system to compete against limited, non-renewable, energy sources due to their high energy density, sustainability, and low cost. Among the main challenges of this technology, researchers are concentrating on reducing the well-known “shuttle effect” that generates the loss and corrosion of the active material during cycling. To tackle this issue, metal-organic frameworks (MOF) are considered excellent sulfur host materials to be part of the cathode in Li-S batteries, showing efficient confinement of undesirable polysulfides. In this study, MIL-88A, based on iron fumarate, was synthesised by a simple and fast ultrasonic-assisted probe method. Techniques such as X-ray diffraction (XRD), Raman spectroscopy, Thermogravimetric Analysis (TGA), Scanning Electron Microscopy (SEM), and N_2_ adsorption/desorption isotherms were used to characterise structural, morphological, and textural properties. The synthesis process led to MIL-88A particles with a central prismatic portion and pyramidal terminal portions, which exhibited a dual micro-mesoporous MOF system. The composite MIL-88A@S was prepared, by a typical melt-diffusion method at 155 °C, as a cathodic material for Li-S cells. MIL-88A@S electrodes were tested under several rates, exhibiting stable specific capacity values above 400 mAh g^−1^ at 0.1 C (1C = 1675 mA g^−1^). This polyhedral and porous MIL-88A was found to be an effective cathode material for long cycling in Li-S cells, retaining a reversible capacity above 300 mAh g^−1^ at 0.5 C for more than 1000 cycles, and exhibiting excellent coulombic efficiency.

## 1. Introduction

The current energy economy based on the demand for non-renewable sources, such as fossil fuels and oil, continues to be a high-risk social problem. The increase in CO_2_ emissions, global warming, and dramatic climate change, are some of the main problems that are taking place and seriously worrying the future of world society [1,2,3]. Therefore, it is vitally important to replace these energy generation systems with others based on cleaner and more environmentally sustainable sources of renewable energy [4,5]. In this area, batteries are currently considered a fundamental energy storage system due to their high efficiency and lifetime, and are being postulated as the key to future development of power system applications [6,7,8].

Lithium-sulfur (Li-S) and lithium-oxygen (Li-O_2_) batteries were proposed as promising high-energy rechargeable systems for emerging applications. Despite the various problems that still present, these two attractive systems exceed the initial expectations of them evolving rapidly, improving the efficiency and achieving high performances, which could lead to these devices to their possible implantation in the development of renewable energy sources, electric vehicles and modern electronics [9,10,11]. Specifically, Li-S batteries are at the forefront in the development of efficient and sustainable high energy systems receiving great interest in recent years [12]. These types of batteries consist of a sulfur cathode, a lithium metal anode, and a polymeric or liquid electrolyte, and are based on the following electrochemical reaction: 16 Li + S_8_ ⇄ 8 Li_2_S [13,14]. The most relevant feature of this type of energy storage system is the high theoretical specific capacity of 1675 mAh g^−1^, and a high theoretical specific energy of 2600 Wh kg^−1^, considering a total conversion which exceeds 5 times the theorical energy supplied by Li-ion based systems [15,16,17]. Apart from these outstanding characteristics, elemental sulfur is a very abundant element in nature, low cost, and environmentally sustainable, while lithium provides advantages such as its low standard potential and light weight [18,19].

The current challenge in these type of systems is mainly focussed on reaching the theoretical values of specific capacity, which is rather complicated because there are several problems derived from practical applications [20]. The dissolution of the lithium sulfur polysulfides (Li_2_S*_x_*) inside the electrolyte generates the main problem of this type of battery, which is called the “shuttle” effect [21]. The soluble polysulfides formed in the cathode are directed towards the anode, reacting with the Li surface forming insoluble species Li_2_S and Li_2_S_2,_ that are continuously deposited at the anode during the charge/discharge cycles [22]. This sequence of unwanted reactions leads to a short life cycle in Li-S batteries, poor efficiency, corrosion of the lithium anode, and a fading of the specific capacity of these systems. Based on this, most research is focussed on the search for various solutions by developing functional materials that make it possible to mitigate this negative effect [23].

In view of novel materials that are attracting enormous attention in recent years, metal-organic frameworks (MOFs) have become one of the most researched areas by the scientific community due to their excellent properties [24]. These types of ordered materials are mainly constructed by metallic ions (clusters) interconnected through multidentate organic ligands (linkers) in networks of one, two, or three dimensions. The high surface area, crystallinity, controllable pore size, flexibility, and functionalisation of the porous surface are some of the main characteristics which determine the versatility of MOFs [25]. Due to their exceptional properties, great interest has been shown in these materials in numerous research areas such as adsorption, catalysis, photocatalysis, luminescence, and electrochemistry [26,27,28,29]. Of fundamental interest in applying porous materials based on MOFs in Li-S batteries is their excellent performance as sulfur hosts, and the confining capacity of the polysulfides generated in the charge/discharge processes, so reducing the undesirable “shuttle” effect [30,31].

Various factors affect the performance of Li-S batteries using a certain MOF as a cathodic host material, such as pore structure, adequate particle size, functional organic linkers, and open metal sites in the metal-organic structure [32]. There have been several works reported where MOF materials are included as components that constitute Li-S batteries as a cathode [33,34], or as a separator [35,36,37]. In most cases, MOF derivatives are used, or the incorporation of additives into the pristine MOF is carried out to improve the conductivity of these energy systems. For example, ZIF-8 is a widely studied material in this technology, showing a different specific capacity depending on the preparation of the composite used as a positive electrode in a range between 400–900 mAh g^−1^ [38,39,40,41].

The application of pristine MOFs as a cathode in Li-S systems is a field of research currently under development, since most studies in recent years have been focussed on the preparation of composites by modifying the initial metal-organic framework with the objective of improving its properties. The main advantage of the use of pristine MOFs in energy storage systems is their high porosity and crystallinity, so that they can be excellent sulfur hosts and confiners of the polysulfides generated in electrochemical cycling without the need for any additional experimental treatment to modify the original characteristics of the network. Some widely known pristine MOFs, such as NH_2_-MIL-53 (Al), MIL-53 (Al), HKUST-1, or even ZIF-8, have been evaluated by Zhou et al., showing remarkable discharge capacities of 332, 347, 286, and 553 mA h g^−1^ after 300 cycles, respectively [42]. However, in most studies on MOF-based sulfur cathodes, less than 100 cycles are normally reported, as is the case with MIL-100/1(Cr) [43,44], MIL-100(V) [45], Ni-MOF-867 [46], and [(CH_3_)_2_NH_2_]_2_[Cd(L)]·5DMF [47].

Herein, MOF MIL-88A as a host material in Li-S batteries is reported for the first time. MIL-88A is a metal-organic framework based on Fe (III) trimers, octahedrally interconnected through the dicarboxylates of fumaric acid [48]. MIL88-A has been considered as an electronic semiconductor material showing this ability for electronic conduction in photocatalytic [49] or in microbial reduction processes [50]. Some investigations of this material have already been reported in the field of Li-ion batteries, showing highly promising performances and improvements in electrochemical activity due to its high porosity which allows greater contact surface between electrode-electrolyte and an increase in the number of active reaction sites [51,52,53,54]. Therefore, in this work, the synthesis of MOF MIL88-A has been carried out by means of a simple, sustainable, and short-time method, assisted by ultrasound. Taking advantage of its textural and morphological properties, this pristine MOF has been used effectively to confine sulfur by the melt-diffusion method. Based on this, the resulting composite is considered a promising candidate as a cathode in Li-S batteries, showing remarkable electrochemical performances, especially during long-cycle testing.

## 2. Materials and Methods

### 2.1. Materials

Sodium hydroxide, iron (III) chloride hexahydrate, fumaric acid, absolute ethanol (99.8%), and *N*,*N*-dimethylformamide (DMF, 99.8%) were obtained from PanReac AppliChem and used as-received. Sublimed sulfur powder (S, VWR Scientific, Hudson, NH, USA) was dried at 45 °C under vacuum overnight. Carbon black Super P (CB, Timcal, Thermo Fisher, Kandel, Germany) and polyvinylidene fluoride (PVDF, Sigma-Aldrich, Merck, Madrid, Spain) were stored at 60 °C. *N*-methyl-2-pyrrolidone (NMP, anhydrous, 99.5%) was supplied by Sigma-Aldrich. Lithium metal (Li, Gelon Lib, Qingdao, China, 15.6 mm diameter and 0.25 mm thick), 1,3-dioxolane (DOL, anhydrous, 99.8%, Sigma-Aldrich) and 1,2-dimethoxyethane (DME, anhydrous, 99.5%, Sigma-Aldrich) were also used as-received but stored under an Ar-atmosphere. Lithium bis(trifluoromethanesulfonyl)imide (LiTFSI, Sigma-Aldrich) and lithium nitrate (LiNO_3_, Sigma-Aldrich) were dried at 120 °C under vacuum for three days. Polyethylene membrane (PE, 25 µm thick, Celgard, Charlotte, NC, USA) was used as a separator and dried at 80 °C under vacuum for 3 h. Carbon cloth Gas Diffusion Layer (GDL, ELAT LT1400W, FuelCellStore, College Station, Texas, USA, 454 µm thick) was used as a substrate.

### 2.2. Synthesis of MIL-88A and MIL-88A@S Composite

The ultrasonic method is a facile and rapid process widely used for the synthesis of MOFs [55,56]. In this case, MIL-88A was prepared using a continuous ultrasonic probe (Branson Sonifier, Danbury, CT, USA, 150) since it has been verified in a previous study that a smaller particle size, and a greater aspect ratio, were obtained with this method compared to an ultrasonic bath [57]. 

The detailed process consisted in the preparation of a 1.25 M ethanolic solution of sodium hydroxide, which was used to dissolve FeCl_3_·6H_2_O (1 mmol) in approximately 10 mL of the DMF:ethanolic solution with a volume ratio of 4.5:1, while maintaining a constant molar ratio (NaOH/Fe = 0.8). In parallel, an equimolar ratio of fumaric acid (1 mmol) was dissolved in 5 mL of DMF. Afterwards, both solutions were mixed and sonicated with a probe for 10 min, using 20 W and 10 kHz in continuous wave mode. Then, the obtained precipitate was centrifuged at 9000 rpm for 10 min and washed in DMF and in ethanol several times. Finally, the sample was recovered and dried under vacuum at 85 °C, overnight, to obtain a porous iron fumarate MOF MIL-88A.

Sulfur can be effectively embedded into the MIL-88A structure by a classical melt diffusion method [58,59]. The as-prepared MIL-88A and elemental sulfur were dried under vacuum and then thoroughly mixed inside an argon-filled glovebox using a mass ratio of 1:1. Then, the mixture was thermally treated in an Ar atmosphere at 155 °C for 20 h. After cooling down, the sulfur solidifies and contracts forming sulfur crystals that bind closely to the host material, finally producing the MIL-88A@S composite.

### 2.3. Characterisation Techniques

X-ray diffraction patterns (XRD) were obtained in a Bruker D8 Discover X-ray diffractometer equipped with a monochromatic Cu K*α* radiation (*λ* = 1.5406 Å). The patterns were recorded within the 5–80° (2*θ*) range, using a step size of 0.04° and 1.05 s per step. Raman spectroscopy was applied with a Renishaw Raman instrument (InVia Raman Microscope) furnished with a Leica microscope using a green laser light excitation source (532 nm). The N_2_ adsorption–desorption measurements were performed with an Autosorb iQ/ASiQwin (Quantachrome Instruments) and the samples were previously degassed. Sample morphology was investigated with a JEOL JSM-7800F scanning electron microscope (SEM). Also, to verify the sulfur content in the MIL-88A@S composite, thermogravimetric analysis (TGA) measurements were carried out using a Mettler Toledo-TGA/DSC under nitrogen atmosphere by heating the samples from 25 to 600 °C at 5 °C min^−1^.

### 2.4. Cathode Preparation and Electrochemical Characterisation

The positive electrodes were prepared by doctor blade casting of a slurry formed by the MIL-88A@S composite (70 wt.%), carbon black (20 wt.%) as a conducting agent, and PVDF (10 wt.%) as a binder in 0.8 mL of *N*-methyl-2-pyrrolidone on a GDL foil. GDL carbon cloth has proven to be an effective current collector in cathodes of Li-S cells [60,61]. The electrode was dried at 80 °C on a heating plate for 3 h and cut into discs of 13-mm diameter with a sulfur loading between 1.0–1.5 mg cm^−2^. Then, the working electrodes were dried under vacuum at 45 °C overnight. 

CR2032-type coin cells were assembled inside a glove box (Ar-filled, M-Braun 150) with discs of Li as negative electrodes. The electrolyte was 1.0 M LiTFSI 1.0 M and 0.4 M LiNO_3_ 0.4 M in DOL:DME (1:1 *v*/*v*) soaked in a porous polyethylene separator, cut into 16-mm diameter discs and dried at 80 °C under vacuum, overnight, prior to use. The addition of LiNO_3_ in the electrolyte causes the oxidation of the polysulfides which become favourable components for the solid electrolyte interphase (SEI), resulting in a decrease in the shuttle effect and preventing parasitic redox reactions between Li and the polysulfides [62]. 

The electrochemical measurements were carried out using an Arbin BT2143 potentiostat–galvanostat system. The working electrodes were galvanostatically cycled between 2.6–1.85 V vs. Li^+^/Li. Specific capacity values are referred to the mass of elemental sulfur in the electrodes. Cyclic voltammetry (CV) curves and electrochemical impedance spectroscopy (EIS) were recorded on an Autolab equipment. CV was performed at a scan rate of 0.1 mV s^−1^ within the 1.7–2.6 V range. Impedance spectra were recorded at the open circuit voltage (OCV) condition and after the 3rd CV cycle, by applying an alternative voltage signal of 10 mV amplitude within the 500 kHz to 0.1 Hz frequency range.

## 3. Results and Discussion

### 3.1. Textural, Morphological and Structural Properties

**Figure 1:**
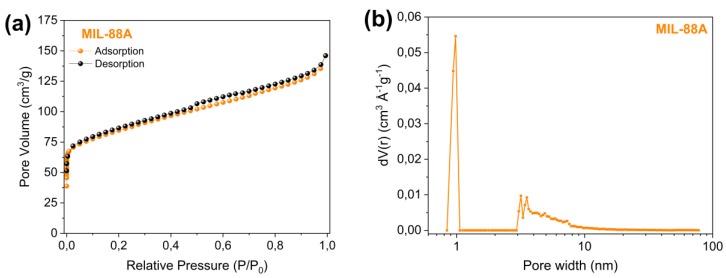
(**a**) N_2_ adsorption-desorption isotherm and (**b**) pore size distribution of MIL-88A.

The continuous probe ultrasound-assisted synthesis of the MOF MIL-88A was successfully performed in a short time. The N_2_ adsorption-desorption isotherm and pore size distribution (obtained by the Density Functional Theory, DFT method) are shown in Figure 1. The textural properties obtained are characteristic for this type of material, showing a high specific surface area (*S*_BET_) of 313 m^2^ g^−1^, pore volume of 0.22 cm^3^ g^−1^, and an average pore width of 9.8 Å, sufficient to serve as a host of the sulfur and polysulfides generated in the charging and discharging in the Li-S systems. These surface area and pore volume values are higher than those reported for the MIL-88A MOF obtained by hydrothermal synthesis [63]. The adsorption-desorption curves for the MIL-88A were a combination of type IV isotherm, with a slight hysteresis loop at relative pressures *P*/*P*_0_ between 0.5–0.8, revealing the presence of mesopores, and type *I* isotherms, with adsorption values in the filling range of micropores at low relative pressures. The analysis by the DFT model showed a wide distribution of pore sizes, comprising a similar proportion of micropores and mesopores. The micropore volume calculated by the t-method (0.08 cm^3^ g^−1^) represents 36% of the total pore volume, confirming the dual meso-microporous character for this material. This interconnected micro-/mesopore system is an ideal candidate to host sulfur, be used as a cathode, and favour the entrapment of lithium polysulfides in Li-S batteries [64].

**Figure 2:**
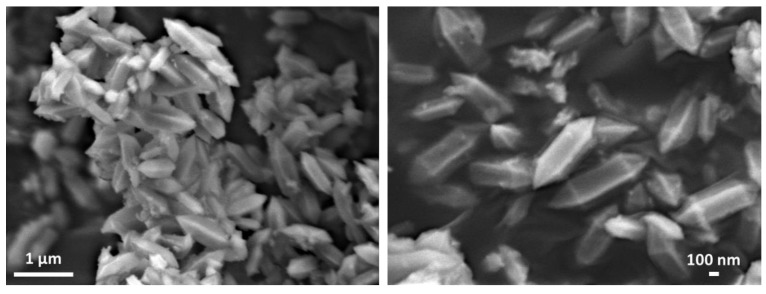
SEM images at different magnifications for MIL-88A MOF.

SEM images of MIL-88A MOF are shown in Figure 2. Its morphology consisted of particles with a well-defined central prismatic portion and pyramidal terminations. It has been reported that ultrasonic assisted synthesis allows for smaller particle size growth of MIL-88A than in conventional solvothermal methods [57]. In this case, the crystal sizes of this MOF were approximately between 900–950 nm long and 200–250 nm wide. This particle shape and size is very suitable and promising to facilitate dispersion and optimal sulfur hosting. The morphology of the sub-micrometric elongated rod-like crystals presented in this MOF obtained by ultrasonic synthesis, is similar to that reported for MIL-88A prepared by different solvo/hydrothermal synthetic methods [56].

**Figure 3:**
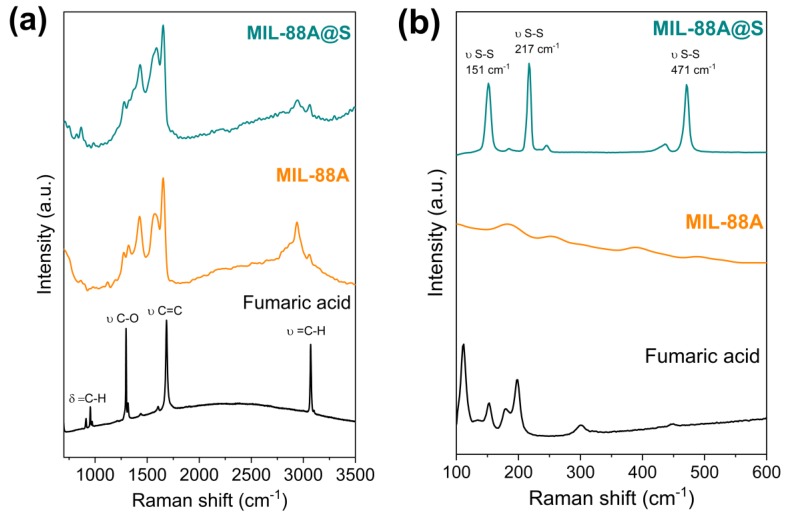
Raman spectra for commercial fumaric acid, MIL-88A and MIL-88A@S registered in shift ranges of 700-3500 (**a**) and 100–600 cm^−1^ (**b**).

Raman spectra shown in Figure 3 confirm the presence of the organic fumarate ligand in MIL-88A and sulfur in the MIL-88A@S composite. In Figure 3a, the double bond of the fumaric acid molecule in MIL-88A and MIL-88A@S was evidenced by the appearance of a band at 3071 cm^−1^, corresponding to the stretching sp^2^ C-H vibrations, and a strong peak at 1687 cm^−1^ associated to the symmetric vibration modes C=C [65]. The bands located at 1580 and 1430 cm^−1^, in the case of fumaric acid, MIL-88A, and MIL-88A@S materials are attributed to the anti-symmetric and symmetric vibrations, respectively, of the carboxylate groups [66,67,68]. The C-O bond was observed as an intense peak centred at 1297 cm^−1^. Additionally, the bending vibrations outside the plane of the bond =C-H were placed at Raman shifts of 970, 953, and 910 cm^−1^. The Raman spectra of MIL-88A and MIL-88A@S show a peak below 3000 cm^−1^ that is attributed to sp^3^ C-H bond vibrations of solvent molecules remaining coordinated to these materials, despite the drying treatment after the synthesis. As shown in Figure 3b, the sulfur contribution in the MIL-88A@S composite was corroborated by the presence of the characteristic S-S peaks in the orthorhombic octahedron α-S_8_ at 471, 217, and 151 cm^−1^ [69,70,71].

**Figure 4:**
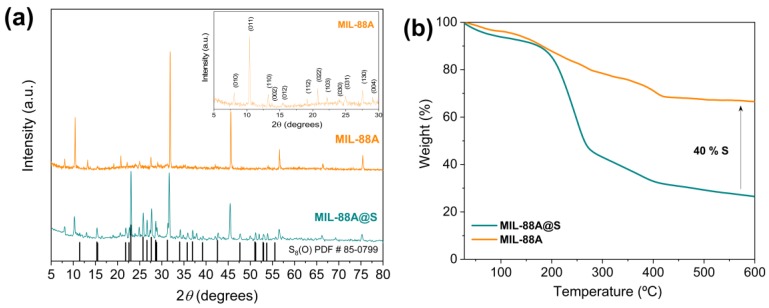
(**a**) XRD patterns of MIL-88A (orange), MIL-88A@S composite (green), and reference data for S_8_ (PDF# 85-0799, black). Inset: XRD patterns of MIL-88A recorded from 5° to 30° (2*θ*); and (**b**) TGA curves of both synthesised samples.

In Figure 4a, the crystallinity of MIL-88A and MIL-88A@S composite is studied by X-ray diffraction (XRD). The inserted image shows the main peaks of the pristine MIL-88A sample, and it can be verified that the position of all of them is in accordance with the structure of this MOF as observed in previous studies [63,72]. On the other hand, the MIL-88A@S composite exhibits well-defined peaks corresponding to the orthorhombic sulfur (PDF # 85-0799), and also the characteristic peaks of the lattice structure of this iron MOF. These results are attributed to the fact that the incorporation of sulfur does not alter the structural properties of the host material. It should be noted that the presence of the signals due to *S* would be justified when a slight excess of sulfur in the composite is demonstrated. This fact indicates that not all sulfur is confined in the porosity of the matrix but that part of this sulfur is deposited on the surface [73]. Moreover, Figure 4b depicts the thermogravimetric stability of the prepared MOF tested at a temperature range of 30–600 °C using an N_2_ flow. There are mainly three regions in the decomposition pattern. In the first step, the weight loss around 100 °C was due to the loss of adsorbed water and the remaining solvent molecules. In addition, the organic ligand was progressively decomposed in a couple of stages, leading to the entire collapse of the organic structure at 420 °C. Hence, the TGA curve of MIL-88A reinforces that the removal of residual solvent molecules from the pores results in a high surface and large pore volume [74]. Furthermore, the amount of sulfur fixed to the MOF structure could be quantified using the TGA curve of the sample MIL-88A@S in an inert atmosphere. In particular, the evaporation of sulfur occurs between 160–400 °C, and finally, the percentage of sulfur assimilated by the host material was 40% by weight.

### 3.2. Electrochemical Properties

**Figure 5:**
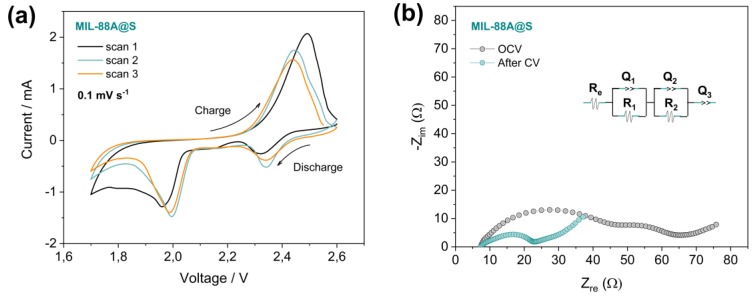
(**a**) CV profiles of Li/LiTFSI-LiNO_3_-DOL:DME/MIL-88A@S battery at room temperature. Scan rate: 0.1 mV s^−1^, voltage range: 1.7–2.6 V; and its corresponding (**b**) EIS measurements in open circuit voltage (OCV) and after the third CV cycle.

The study of the electrochemical performance of the Li-S battery using the MIL-88A@S composite was carried out by cyclic voltammetry (CV), electrochemical impedance spectroscopy (EIS), and galvanostatic measurements (GCD), and was conducted to investigate the redox process of the active material. The cyclic voltammograms of the Li/LiTFSI-LiNO_3_-DOL:DME/MIL-88A@S cell containing lithium metal anodes, liquid electrolyte, and composites cathodes of MOF and sulfur, are shown in Figure 5a. During the first scan, two cathodic peaks are observed at 2.32 V and 1.95 V respectively, and an anodic peak at 2.49 V. In this first discharge, the broad reduction peak could be due to the fact that the electrochemical reaction needs to overcome the absorbing energy between S and the conductive matrix, as well as the low dissolution capacity of the polysulfide in the liquid electrolyte [75]. During successive scans, the peaks appear displaced; in the discharge process at 2.33 V and 1.99 V, and during the oxidation process at lower values, around 2.44 V. After the shift of the potentials between the first CV curve and the remaining ones, a smaller potential gap between the anodic and cathodic peak takes place, resulting in a lower polarisation and resistance within the cell. This fact is corroborated with the EIS measurements of the positive electrode, consisting of a mixture of sulfur and an MOF based material. Figure 5b shows the Nyquist plots of the impedance spectroscopy test performed before and after the CV cycles. In both cases, the equivalent circuit of the cell would be R_e_(R_1_Q_1_)(R_2_Q_2_)Q_3_, where the electrolyte resistance (R_e_) is similar in OCV and after the CV cycles, with values of 7.28 Ω and 7.22 Ω, respectively; the medium-high frequency semicircles (R_i_Q_i_) are related to the electrode/electrolyte interface, and consequently, to the formation of the SEI; and the low-frequency pseudo-capacitance (Q_3_) represents the semi-infinite Li^+^ diffusion or capacitive behaviour of the cell [76]. In addition, a marked decrease in global resistance values can be observed from 57.23 Ω, at the OCV, to values of 23.15 Ω after the cyclic voltammetry tests. This process, that entails a decrease in the resistance of the cell, indicates that electrode activation cycles would be necessary, and even, in some cases, the voltage window used for galvanostatic cycling at high rates should be modified [77].

**Figure 6:**
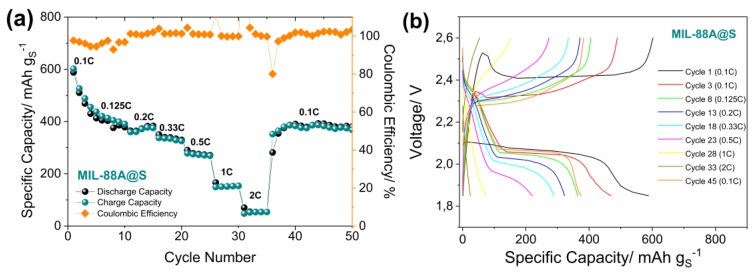
(**a**) Rate capability cycling behaviour performed at C/10, C/8, C/5, C/3, C/2, 1 C, and 2 C (1C = 1675 mA g^−1^) at room temperature, and (**b**) corresponding voltage profiles of a Li/S cell using the LiTFSI-LiNO_3_-DOL:DME electrolyte and MIL-88A@S electrode. Voltage limits 1.85 V–2.6 V.

The galvanostatic measurements of the Li/LiTFSI-LiNO_3_-DOL:DME/MIL-88A@S cells were carried out by cycling at various currents as well as at a constant C-rate of 0.5 C. The rate capability test (Figure 6a,b) for the MIL-88A@S composite provided an initial capacity of 600 mAh g^−1^ that was stabilized over the first five cycles, displaying an average discharge capacity of 482 mAh g^−1^ at 0.1 C. This maximum capacity value gradually decreased to about 395, 374, 333, 274, 152, and 55 mAh g^−1^, at 0.125 C, 0.2 C, 0.33 C, 0.5 C, 1 C, and 2 C, respectively, as demonstrated by the cycling trends of Figure 6a. When the current rate was restored to 0.1 C, the electrode recovered a capacity value of about 375 mAh g^−1^. In addition, the coulombic efficiency of the cell is represented on the right axis and at low current densities (0.1 and 0.125 C), values thereof close to 95% can be observed, while for the remaining cycling rates, these values are close to 100%. This phenomenon is seen more clearly in Figure 6b, where the charge and discharge profiles during the first cycles are not well defined. Additionally, charge profiles of the 1^st^ and 2^nd^ cycles showed a high polarisation at the beginning of cycling as previously indicated in the first cycle of the CV, which could be caused by the greater resistance that the cell presents until the SEI is formed, as previously observed for MOFs as sulfur hosts in Li–S batteries [78].

**Figure 7:**
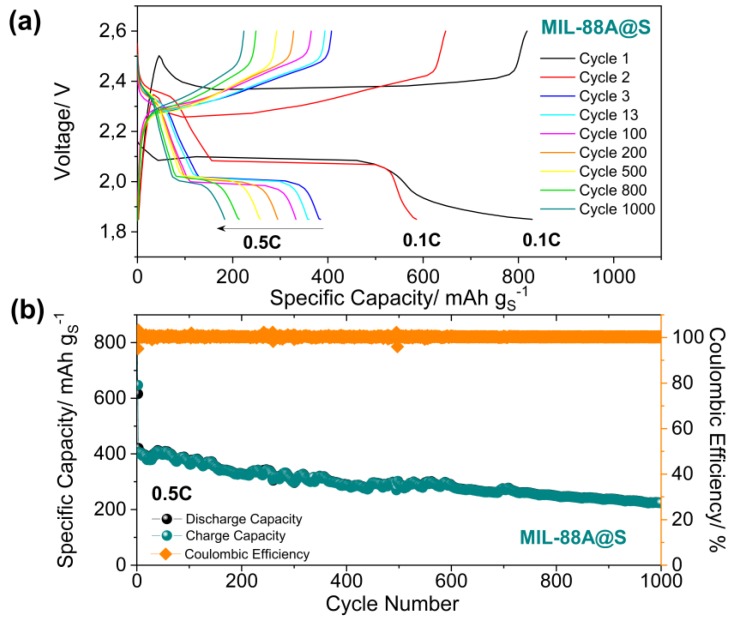
(**a**) The discharge and charge profiles of a Li-S cell using a constant rate of 0.5 C (1 C = 1675 mA g^−1^) within a potential range of 1.85-2.6 V vs. Li^+^/Li, and (**b**) the delivered capacity from the discharge and charge and coulombic efficiency vs. the cycle number.

In order to avoid these harmful effects, the Li/LiTFSI-LiNO_3_-DOL:DME/MIL-88A@S cell has been studied by a long-term cycling at a moderate constant current of 0.5 C (Figure 7), after two activation cycles performed at 0.1 C. As can be seen, two plateaus at 2.35 V and 2.0 V are clearly observed during the discharge process at 0.5 C, which correspond to the formation of long chain lithium polysulfides (Li_2_S*_x_*, 4 ≤ *x* ≤ 8), and short-chain lithium polysulfides (such as Li_2_S_2_ and Li_2_S), respectively, and which correspond to the potential values of the cathode peaks obtained in the cyclic voltammetry measurements (Figure 5a). Such a discharge profile is typical for sulfur cathodes [79]. From the third cycle, the cell delivers an initial discharge capacity of about 400 mAh g^−^^1^, and 200 mAh g^−1^ after 1000 cycles, revealing a slow decay of 0.05% per cycle, possibly attributed to the progressive loss of active material caused by the irreversible formation of short-chain lithium polysulfides [80]. More strikingly, after more than 1000 cycles at 0.5 C, the Li/LiTFSI-LiNO_3_-DOL:DME/MIL-88A@S cell is still working, providing 100% coulombic efficiency after activation cycles and exhibiting remarkable cycling stability. 

Few previous studies have reported the long-term cyclability for simple MOF@S cathodes in Li-S batteries. Zhou et al. [42] demonstrated that Li-S cells, based on MOFs such as ZIF-8, MIL-53 (Al), NH_2_-MIL-53 (Al), and HKUST-1, reach capacities of 550, 345, 330, and 285 mAh g^−1^ respectively, after 300 cycles at 0.5 C rate. However, although the composites contained 50 wt.% sulfur, the electrodes had to be prepared with 30 wt.% conductive agent (CB), reducing the active composite content in the cathode. To the best of our knowledge, only Bai et al. [33] have reported MOF-based cathodes with ultra-high cyclability. Sulfur composites based on ZIF-8, ZIF-67, and HKUST-1, have delivered specific capacities of 170, 150, and 250 mAh g^−1^ respectively, after 1000 cycles at 0.2 C rate. Two disadvantages are found compared to the MIL-88A@S presented in our work: the need to previously synthesise sulfur nanoparticles, and the lower active sulfur content in composites (below 30 wt.% *S*). Therefore, the MIL-88A MOF is presented for the first time as an effective sulfur host for Li-S cells, with an appropriate sulfur content in the cathode and remarkable stability in long-term cyclability.

## 4. Conclusions

In summary, an iron metal organic framework (MIL-88A) has been prepared by fast ultrasound-assisted synthesis. XRD and textural analyses have confirmed that the MIL-88A material exhibited a high crystallinity, specific area and pore volume, suitable to be used as a matrix to host sulfur in Li-S batteries. Sulfur was incorporated by the melt-diffusion method at 155 °C because the conventional composites preparation methods for carbonaceous matrices were unsuccessful. In fact, the main problems initially observed in the synthesis of composites were the loss of the morphology by the use of ball milling, or the deterioration of the MOF due to the use of certain solvents. Next, the positive electrode was directly prepared using the primitive MOF, without calcining and without adding any additional additives to those commonly used for the manufacture of electrodes (CB and PVDF). MIL-88A@S composite demonstrated good storage capacity at high current densities and a brilliant electrochemical response during prolonged cycling to 1000 cycles, supplying an average specific capacity of 300 mAh g^−1^ at a sizeable rate of 0.5 C. The results thus suggest that this composite material could be a promising component for lithium-sulfur batteries. Besides, the appealing property of these cathodes is the excellent long-term cycling stability, considering that it works directly with the primitive MOF.
